# Management of Spasticity in Children with Cerebral Palsy

**Published:** 2014-05-09

**Authors:** Alireza Shamsoddini, Susan Amirsalari, Mohammad-Taghi Hollisaz, Alireza Rahimnia, Amideddin Khatibi-Aghda

**Affiliations:** 1 Exercise Physiology Research Center; 2 Department of Pediatric Neurology; 3 Department of Orthopedic Surgery, Baqiyatallah University of Medical Sciences, Tehran, Iran

**Keywords:** Cerebral Palsy, Intrathecal Baclofen, Occupational Therapy, Physical Therapy, Rhizotomy, Spasticity

## Abstract

Cerebral palsy is the most common cause of spasticity and physical disability in children and spasticity is one of the commonest problems in those with neurological disease. The management of spasticity in children with cerebral palsy requires a multidisciplinary effort and should be started as early as possible. There are a number of treatments available for the management of spasticity. This article reviews the variety of options available for the clinical management of spasticity.

## Introduction

Cerebral palsy (CP) is defined as a clinical syndrome characterized by a persistent disorder of posture or movement due to a non-progressive disorder of the immature brain^[^^1^^]^. The prevalence of CP is 2 to 2.5 per 1,000 live births^[^^2^^]^ and its incidence may be increasing secondary to improved care in neonatal intensive care units and improved survival of low birth-weight infants^[^^3^^]^. Most children with CP will have spasticity as the main motor disorder and it can be classified either according to which body areas is affected: hemiplegia, diplegia, tetraplegia, or the movement disorder type: spastic, athetoid, ataxic and hypotonic cerebral palsy^[^^2^^,^^3^^,^^5^^]^. Spasticity is a major challenge for rehabilitation of children with cerebral palsy. Spasticity can prevent or hamper function, cause pain, disturb sleep, cause unnecessary complications and present major difficulties for care workers^[^^6^^]^.

 The paper is based on literature searches in PubMed, ISI Web of Science and Google Scholar using the key phrases «management of spasticity and cerebral palsy», with the emphasis on clinical studies. Our assessments also rest on our own clinical experience and research at Baqiyatallah Hospital.

***Literature Review: ***There are epidemiological, clinical and review studies about management of spasticity in children with cerebral palsy.

***Definitions of Spasticity***

Most physicians and therapists working with children with cerebral palsy probably feel that they can recognize spasticity when they see or feel it^[^^1^^]^. Spasticity is defined as a velocity dependent increased resistance to passive muscle stretch, or alternatively as inappropriate involuntary muscle activity associated with upper motor neuron paralysis^[^^7^^,^^8^^]^. Spasticity can result in functional problems with daily living activities (ADL) such as gait, feeding, washing, toileting and dressing^[^^9^^]^. Over time, spasticity may also cause problems, such as muscle pain or spasms, trouble moving in bed, difficulty with transfers, poor seating position, impaired ability to stand and walk, dystonic posturing muscle, contracture leading to joint deformity, bony deformation, joint subluxation or dislocation and diminished functional independence. Contractures occur when there is loss of joint motion due to structural changes in the muscles, ligaments and tendons surrounding the joint. Shortening and stiffness of the soft tissues make the joint resistant to stretching and prevent normal movement^[^^4^^,^^5^^,^^10^^-^^12^^]^. However, spasticity is a benefit for children with cerebral palsy. Increased tone may be useful for the child. It helps to keep the legs straight, thereby supporting the child’s weight against gravity. The child with increased tone in trunk extensors may stand and take a few steps. Spasticity may help preserve muscle bulk and bone density (Table 1)^[^^11^^]^. The extent and type of spasticity can fluctuate widely according to position of head and limbs, fatigue, stress and mood of children. One limb may have one pattern of spasticity whilst another may have a different pattern^[^^6^^]^.

**Table 1:** Adverse and beneficial effects of Spasticity

	**Effects of spasticity**
**Negative effects**	Abnormal posture
	Difficulty in hygiene and dressing
	Difficulty in movements
	Difficulty in sitting and transfers
	Inhibits muscle growth
	Joint subluxation or dislocation
	Leads to contractures
	Masks contraction in the antagonist
	Muscle Pain
	Pressure sores
	Shortening and stiffness of the soft tissues
**Positive effects**	Extensor tone in the limbs help standing
	Preserve bone density
	Preserve muscle bulk

***Causes of Spasticity***

Spasticity in children can result from any disease process that affects the upper motor neuron within the central nervous system. Injury to the upper motor neuron decreases cortical input to the descending reticulospinal and corticospinal tracts, which causes weakness, loss of motor control, and reduction in the number of voluntarily active motor units. The reduction of these descending tracts removes the normal inhibition of the reflex arcs within the grey matter of the spinal cord, leading to a hyperactive reflex arc and spasticity^[^^13^^]^. While in certain cases there is no identifiable cause, typical causes include problems in intrauterine development (e.g. exposure to radiation, infection), asphyxia before birth, hypoxia of the brain, birth trauma during labor and delivery, and complications in the prenatal period or during childhood. Infections in the mother, low birth weight (less than 2.0 Kg) is a risk factor for CP. Also, between 40 and 50% of all children who develop CP were born prematurely. Premature infants are vulnerable, in part because their organs are not fully developed, increasing the risk of hypoxic injury to the brain that may manifest as cerebral palsy^[^^14^^]^.

***Measuring Spasticity***

The diagnosis of spasticity in children with CP requires a complete physical examination, with ancillary testing as needed. The physical examination should focus on motor power, muscle tone, active and passive range of motion of joints, sensation, deep tendon reflexes, station (pelvic and leg alignment while standing, if there is a possibility), presence of upper and lower limbs deformity, spinal alignment^[13]^. Mechanical instruments and electrophysiological techniques can also be used to assess spasticity. Mechanical instruments measuring the resistance of the muscle to passive stretch and electrophysiological measures showing the hyper excitability of the stretch reflex are used only for research purposes^[^^15^^]^. One of most important tests in rehabilitation for physical examination of spasticity is the Ashworth scale (Table 2). Always test the patient while he or she is in a relaxed supine position.

**Table 2:** Ashworth Scale of Muscle Tone

**Ashworth Scale**	**Degree of Muscle Tone**
**1**	No increase in tone
**2**	Slight increase in tone, “catch” when limb is moved
**3**	Marked increase in tone, passive movements difficult
**4**	Considerable increase in tone, passive movements difficult
**5**	Affected part is rigid in flexion or extension

Passively move the joint rapidly and repeatedly through the available range of motion and grade the resistance using the definitions^[^^8^^,^^12^^,^^16^^]^. Individual assessment, prefer- ably with the aid of video clips from before and after treatment, may be useful for assessing effectiveness. One important parameter will always be whether the aims of the treatment were fulfilled.

 Management of spasticity is a major challenge to treatment team. Various forms of therapy are available to people living with cerebral palsy as well as caregivers and parents caring for someone with this disability. They can all be useful at all stages of this disability and are vital in a CP person's ability to function and live more effectively^[^^17^^]^. There is no standardized approach to spasticity management of cerebral palsy. But adequate assessment of the specific impairments causing disability is necessary for appropriate interventions to be instituted^[^^18^^]^. The treatment strategy depends on the degree of functional failure caused by the spasticity and its location. In general, treatment options for management of spasticity in children with cerebral palsy include oral medications, physical and occupational therapy, splinting and casting, chemodenervation with botulinum toxin or phenol, selective dorsal rhizotomy, intrathecal baclofen, and orthopedic surgery^[^^4^^-^^6^^,^^8^^,^^10^^,^^11^^,^^17^^,^^18^^]^.

***Oral Medications***

Oral medications are a systemic, rather than focal, treatment for spasticity in children with cerebral palsy. Oral medications commonly used in children are baclofen, diazepam, clonazepam, dantrolene and tizanidine^[^^19^^]^.

***Botulinum Toxin***

Botulinum toxin (BT) injection is now an established first-line treatment for focal spasticity^[^^10^^,^^12^^,^^20^^-^^22^^]^. Botulinum toxin type A produces dose-related weakness of skeletal muscle by impairing the release of acetylcholine at the neuromuscular junction. This partially interrupts muscle contraction making the muscle temporarily weaker^[^^20^^-^^22^^]^. Muscles commonly treated with BT include the gastrocnemius-soleus complex, hamstrings^[^^10^^,^^12^^]^, hip adductors and flexor synergy muscles of the upper extremity^[^^21^^,^^22^^]^. Intramuscular injections can be localized by surface landmarks, electromyography stimulation, and/or ultrasound^[^^20^^,^^22^^]^. Following injection, muscle relaxation is evident within 48 to 72 hours and persists for a period of 3 to 6 months^[^^23^^]^. Botox injection can help improve a child’s ability to walk or use hands and allow for a better fitting orthotics by reducing spasticity. Therapists can take advantage of the time when an overly powerful muscle is weakened to work on strengthening the muscle on the opposite side of the joint (antagonist). Sometimes, casting of the involved extremity is done after the injection to increase the stretch of the tight muscle^[^^10^^,^^12^^,^^20^^-^^22^^]^.

***Intrathecal Baclofen***

Intrathecal baclofen (ITB) was approved for the treatment of spasticity of cerebral origin in 1996. ITB is a surgically implanted system used to control spasticity by infusing baclofen directly into the spinal canal and around the spinal cord^[^^24^^]^. Baclofen inhibits spasticity by blocking excitatory neurotransmitters in the spinal dorsal horn. ITB maximizes the dose delivered to spinal receptors and minimizes the side effects associated with oral baclofen^[^^25^^]^.

***Selective dorsal rhizotomy***

Selective dorsal rhizotomy (SDR) derives from late 19th century procedures for spasticity. SDR is a neurosurgical procedure that involves partial sensory deafferentation at the levels of L1 through S2 nerve rootlets^[^^26^^]^. After a series of tone management with rehabilitation punctuated with botulinum toxin injections, the child would probably be around 4 to 5 years old and SDR can be considered. A suitable candidate for selective dorsal rhizotomy is typified by 1) spasticity is still a problem 2) good strength of lower limbs and trunk muscles 3) able to stand straight with good alignment 4) intellectually good enough for carrying out training^[^^27^^]^.

***Splinting, Casting and Orthoses***

**Fig. 1:**
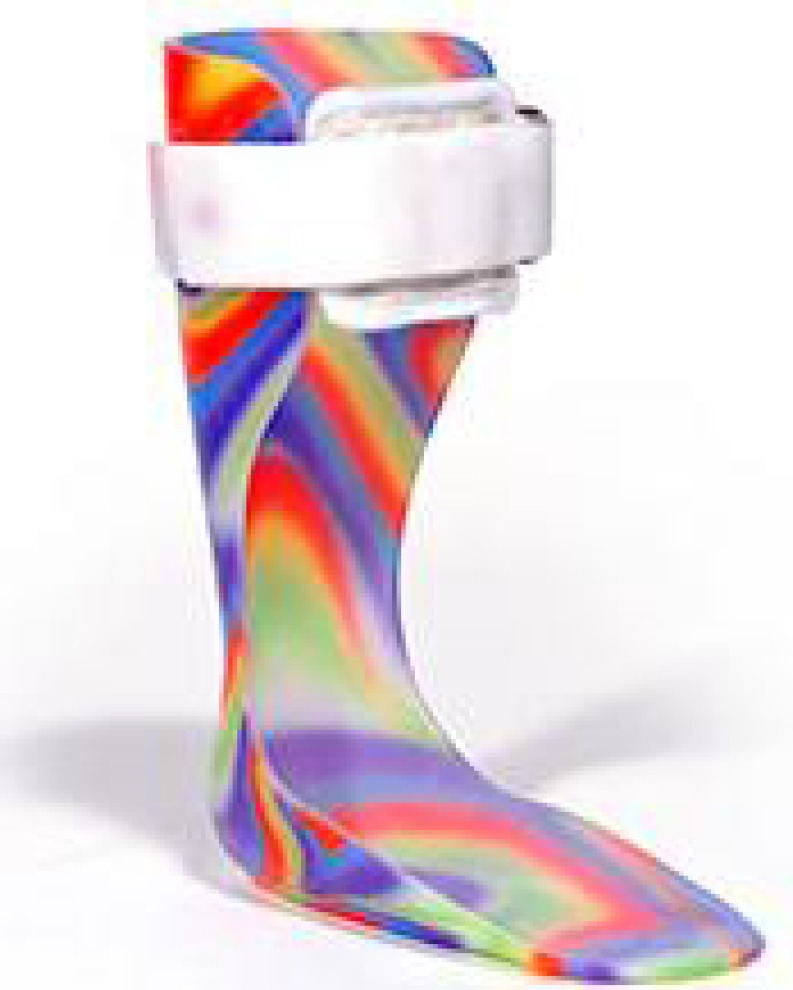
Ankle-foot orthosis (AFO)

Casts, splints, and orthoses are all devices that are designed to keep the body in a certain position. These devices are used to prevent or correct deformities in the spastic limb and/or to help children with cerebral palsy overcome activity limitations, such as difficulties with standing and walking^[^^28^^,^^29^^]^ and serial casting can improve the range of movement in a joint that is already contracted^[^^6^^]^. Serial casting is an intervention practice that is becoming more commonly used in occupational therapy practice, in addition to other treatment modalities/protocols for children with cerebral palsy to manage spasticity and related contractures^[^^30^^]^. Serial casting is based on the premise that shortened muscles maintain the plasticity for lengthening. Providing a prolonged stretch offers biomechanical benefits and inhibits spasticity. But there is a difference between inhibitive casting and serial casting. in inhibitive casting only a single static cast is used and the purpose is to reduce tone rather than lengthen muscle, thereby improving function^[^^31^^]^. The most common type of orthosis is the ankle-foot orthosis (AFO). AFOs are typically designed to limit unwanted ankle movements, specifically ankle plantar flexion (foot pointed toward the ground) (Fig. 1). AFOs can be fixed (to block ankle movement) or articulating (to allow for some movement at the ankle)^[^^32^^]^. Preventing plantar flexion through the use of AFOs has been found to improve walking efficiency in children with spastic diplegic cerebral palsy^[^^33^^]^ and in children with hemiplegic cerebral palsy. When AFO use is compared to barefoot walking, the children's walking patterns are better when wearing AFOs^[^^34^^]^. For children with cerebral palsy who tend to walk on their toes, AFOs have been shown to improve their ability to move from sit to stand. However, children with cerebral palsy who are able to stand on a flat foot did not benefit from AFOs for moving from sit to stand as the AFOs tended to slow them down^[^^35^^]^. AFOs have also been shown to affect how much energy children with cerebral palsy use to walk. One study found that children with spastic diplegic cerebral palsy had lower oxygen needs during walking when they wore hinged AFOs^[^^36^^]^.

***Orthopedic Surgery***

Orthopedic surgery is no option for managing spasticity. Instead, it is used to help correct the secondary problems that occur with growth in the face of spastic muscles and poor motion control. Those problems include muscle shortening, joints contractures and bony deformities^[^^37^^]^.

***Occupational Therapy and Physical Therapy***

Occupational therapy (OT) and physical therapy (PT) are a fundamental part of spasticity management. Muscle overactivity produces muscle shortening and muscle shortening increases spindle sensitivity. Muscle contracture and stretch sensitive muscle overactivity are intertwined. Therefore rehabilitation and physical treatments aimed at lengthening the overactive muscles are fundamental. Address both shortening and overactivity^[^^38^^]^. There are a number of different dynamic Occupational and Physical therapy approaches, including the Bobath technique^[^^4^^,^^5^^]^, Sensory integration therapy^[^^5^^]^, poprioceptive neuromuscular facilitation^[^^39^^]^ and the Brunnstrom technique^[^^40^^]^. Consider applying various techniques such as ice (cold), heat, positioning, stretching exercises and use of orthotic devices for these purposes. Cold inhibits spastic muscles, but the effect is short-lived, perhaps outlasting the application of the cold by about half an hour^[^^41^^]^. Paradoxically, heat is also used for relaxation of a spastic muscle^[^^42^^]^. Position the child to stretch the spastic muscles and decrease the sensitivity of the stretch reflex and the brain stem reflexes that trigger spasticity. Also, the therapists should teach these positions to the family so that the child lies and sits this way most of the time at home^[^^43^^]^. Massage and stretching muscles may prevent contractures and promote muscle growth^[^^44^^-^^46^^]^. Spasticity decreases with slow and continuous stretching^[^^47^^]^. This effect lasts from 30 minutes to 2 hours. Use stretching exercises before bracing and serial casting to obtain the necessary joint position^[^^44^^,^^46^^,^^47^^]^. Also, Orthoses are generally used in conjunction with occupational therapy and physical therapy with the aims of increasing muscle length (through providing a prolonged stretch), breaking up mass patterns of movement and improving biomechanics and stability^[^^48^^-^^51^^]^. Muscle relaxation after stretching exercises lasts for a short period of time. For longer duration the stretch on the muscle should be maintained for several hours every day. This is possible with the use of rigid splints or serial casting^[^^17^^,^^49^^,^^52^^]^.

## Conclusion

The management of spasticity following a cerebral palsy is complex and is a major challenge to treatment team. Initial management should focus on the elimination of externally exacerbating causes. If the spasticity interferes with function, causes pain, and produces deformity, then clear treatment goals should be established. 

 There is not a standardized approach. The treatment needs to be evidence-based and depends on the degree of functional failure caused by the spasticity and its location. This management often requires a variety of different approaches including oral medications, but botulinum toxin, intrathecal baclofen, occupational and physical therapy and often surgical interventions such as selective dorsal rhizotomy and orthopedic surgery.

## References

[1] Mutch L, Alberman E, Hagberg B (1992). Cerebral palsy epidemiology: where are we now and where are we going?. Dev Med Child Neurol.

[2] Reeuwijk A, Van Schie PEM, Becher JG (2006). Effects of botulinum toxin type A on upper limb functions in children with cerebral palsy: a systematic review. Clin Rehabil.

[3] O’Shea TM, Preisser JS, Klinepeter KL (1998). Trends in mortality and cerebral palsy in a geographically based cohort of very low birth weight neonates born between1982 to 1994. Pediatrics.

[4] Shamsoddini AR (2010). Comparison between the effect of neurodevelopmental treatment and sensory integration therapy on gross motor function in children with cerebral palsy. Iran J Child Neurology.

[5] Shamsoddini AR, Hollisaz MT (2009). Effect of sensory integration therapy on gross motor function in children with cerebral palsy. Iran J Child Neurology.

[6] Michael RB (1998). Management of spasticity. Age Ageing.

[7] Goldstein EM (2001). Spasticity management: an overview. J Child Neurol.

[8] Sanger TD, Delgado MR, Gaebler-Spira D (2003). Classification and definition of disorders causing hypertonia in childhood. Pediatrics.

[9] Lundy C, Lumsden D, Fairhurst C (2009). Treating complex movement disorders in children with cerebral palsy. Ulster Med J.

[10] Amirsalari S, Dalvand H, Dehghan L (2011). The efficacy of botulinum toxin type A injection in the hamstring and calf muscles with and without serial foot casting in gait improvement in children with cerebral palsy. Tehran Uni Med J.

[11] Meythaler JM (2001). Concept of spastic hypertonia. Phys Med Rehabil Clin N Am.

[12] Dalvand H, Dehghan L, Feizy A (2012). The effect of foot serial casting along with botulinum toxin type-a injection on spasticity in children with cerebral palsy. J Kerman Uni Med Sci.

[13] Mandigo CE, Anderson RC (2006). Management of childhood spasticity: a neurosurgical perspective. Pediatr Ann.

[14] Beukelman DR, Mirenda P (1999). Augmentative and Alternative Communication: Management of Severe Communication Disorders in Children and Adults.

[15] Berker N, Yalçin S (2010). The HELP Guide to Cerebral Palsy.

[16] Ashworth B (1964). Preliminary trial of carisoprodol in multiple sclerosis. Practitioner.

[17] Tilton AH (2004). Management of spasticity in children with cerebral palsy. Semin Pediatr Neurol.

[18] Matthews DJ, Balaban B (2009). Management of spasticity in children with cerebral palsy. Acta Orthop Traumatol Turc.

[19] Chung CY, Chen CL, Wong AM (2011). Pharmacotherapy of spasticity in children with cerebral palsy. J Formos Med Assoc.

[20] Kinnett D (2004). Botulinum toxin A injections in children: technique and dosing issues. Am J Phys Med Rehabil.

[21] Jefferson RJ (2004). Botulinum toxin in the management of cerebral palsy. Dev Med Child Neurol.

[22] Yang TF, Fu CP, Kao NT (2003). Effect of botulinum toxin type A on cerebral palsy with upper limb spasticity. Am J Phys Med Rehabil.

[23] Russman BS, Tilton A, Gormley ME Jr (1997). Cerebral palsy: a rational approach to a treatment protocol, and the role of botulinum toxin in treatment. Muscle Nerve Suppl.

[24] Roche N, Even-Schneider A, Bussel B (2007). Management of increase in spasticity in patients with intrathecal baclofen pumps. Ann Readapt Med Phys.

[25] Mess SA, Kim S, Davison S (2003). Implantable baclofen pump as an adjuvant in treatment of pressure sores. Ann Plast Surg.

[26] Park TS, Rengachery SS, Wilkins RH (1994). Selective dorsal rhizotomy for the spasticity of cerebral palsy. Neurosurgical Operative Atlas.

[27] Chan NNC (2011). Physiotherapy in Spasticity Management for Children with Cerebral Palsy. Hong KongMed Bulletin.

[28] Christopher M (2002). Orthotic Management of Children with Cerebral Palsy. JPO Online Library.

[29] Teplicky R, Law M, Russell D (2002). The effectiveness of casts, orthoses, and splints for children with neurological disorders. Infants Young Child.

[30] Shashi, Das SP, SVNIRTAR, Gursimrat SC (2011). Efficacy of occu-pational therapy and serial casting in lower limbs of cerebral palsy children administered with phenol nerve block. J Clin Orthop Trauma.

[31] Hinderer KA, Harris SR, Purdy AH (1988). Effects of 'tone-reducing' vs. standard plaster-casts on gait improvement of children with cerebral palsy. Dev Med Child Neurol.

[32] Gok H, Kucukdeveci A, Altinkaynak H (2003). Effects of ankle-foot orthoses on hemiparetic gait. Clin Rehabil.

[33] Crenshaw S, Herzog R, Castagno P (2000). The efficacy of tone-reducing features in orthotics on the gait of children with spastic diplegic cerebral palsy. J Pediatr Orthop.

[34] Buckon CE, Sienko Thomas S, Jakobson-Huston S (2001). Comparison of three ankle-foot orthosis configurations for children with spastic hemiplegia. Dev Med Child Neurol.

[35] Wilson H, Haideri N, Song K (1997). Ankle-foot orthoses for perambulatory children with spastic diplegia. J Pediatr Orthop.

[36] Maltais D, Bar-Or O, Galea V (2000). Use of orthoses lowers the O2 cost of walking in children with spastic cerebral palsy. Med Sci Sports Exerc.

[37] Gage J, Gormley Jr M, Krach L (2004). Managing spasticity in children with cerebral palsy requires a team approach. a pediatric perspective. Gillette Children Spatiality Health Care.

[38] Dodd K, Imms C, Taylor NF (2010). Physiotherapy and Occupational Therapy for People with Cerebral Palsy: A Problem-Based Approach to Assessment and Management.

[39] Levitt S (1966). Proprioceptive neuromuscular facilitation techniques in cerebral palsy. Physiotherapy.

[40] Hastings-Smith R, Sharpe M (1994). Brunnstrom therapy: is it still relevant to stroke rehabilitation?. Physio Theory Pract.

[41] Price R, Lehmann JF, Boswell-Bessette S (1993). Influence of cryotherapy on spasticity of the human ankle. Arch Phys Med Rehabil.

[42] Lehmann JF, deLateur BJ, Lehman JF (1982). Therapeutic Heat. Therapeutic Heat and Cold.

[43] Hinderer SR, Dixon K (2001). Physiologic and clinical monitoring of spastic hypertonia. Phys Med Rehabil Clin N Am.

[44] Rassafiani M, Sahaf R (2011). Hypertonicity in children with cerebral palsy: a new perspective. IRJ.

[45] Dalvand H, Dehghan L, Feizy A (2009). Effect of the Bobath technique, conductive education and education to parents in activities of daily living in children with cerebral palsy in Iran. Hong Kong J Occup Ther.

[46] Cusick A, McIntyre S, Novak I (2006). A comparison of goal attainment scaling and the Canadian Occupational Performance Measure for paediatric rehabilitation research. Dev Neurorehabil.

[47] Dalvand H, Dehghan L, Feizi A (2013). The impacts of hinged and solid ankle-foot orthoses on standing and walking in children with spastic diplegia. Iran J Child Neurol.

[48] Gracies JM (2001). Pathophysiology of impairment in patients with spasticity and the use of stretch as a treatment of spastic hypertonia. Phys Med Rehabil Clin N Am.

[49] David A, Alan L (2006). Orthoses in the Management of Spasticity in the Lower Limb. ACNR.

[50] Pirpiris M, Graham HK, Barnes MP, Johnson GR (2001). Management of spasticity in children. Upper motor neuron syndrome and spasticity: Clinical management and neurophysiology.

[51] Colangelo C, Kramer P, Hinojosa J (1999). Biomechanical frame of reference. Frames of Reference for Pediatric Occupational Therapy.

[52] Khalid AM, Sabahat AW (2009). Management of spastic cerebral palsy in the UAE: An overview. ACNR.

